# Study protocol - assessing parkrun for walking rehabilitation for people living with, and beyond, cancer: acceptability, adherence, social support and physical function

**DOI:** 10.1186/s13102-024-00882-w

**Published:** 2024-04-19

**Authors:** Suzanne Broadbent, Robert Buhmann, Yuri Kriel, Sonja Coetzee, Christian Jones, Michelle Morris, Hattie H Wright

**Affiliations:** 1 School of Health, University of the Sunshine Coast, 4556 Sippy Downs, QLD Australia; 2 School of Law and Society, University of the Sunshine Coast, 4556 Sippy Downs, QLD Australia; 3 Sunshine Coast University Private Hospital, 4575 Birtinya, QLD Australia

**Keywords:** Cancer, Parkrun, Walking, Acceptability, Physical function, Wellness, Diet

## Abstract

**Introduction:**

Due to a variety of barriers, the majority of cancer survivors do not do enough physical activity to meet current recommendations. This study will assess the feasibility of participation in parkrun walk-run events as a novel mode of community rehabilitation exercise.

**Methods:**

This protocol describes a single-arm intervention study with participants acting as their own controls. The study accepts adults diagnosed with any type of cancer, undergoing treatment or in remission. Participants must be able to walk and have medical clearance to exercise. A sample of 100 participants will be recruited across the Sunshine Coast over two years. Data will be collected over 9-months at 4 time points: Baseline (T1); after 4-weeks of usual daily activities and cancer management prior to parkrun participation(T2); after a 6-month parkrun intervention (T3); at 2-month follow-up (T4). The primary objectives are to assess the acceptability of, and adherence to, parkrun as rehabilitation exercise. Secondary outcomes include wellness, health-related quality of life, anxiety, depression, mood, physical function, parkrun metrics, dietary intake, and diet and exercise behaviour.

**Conclusion:**

This study will be the first to examine the long-term effects of parkrun as a cancer rehabilitation modality with regard to physical function, psychosocial outcomes and dietary intake.

**Trial registration:**

Australian and New Zealand Clinical Trials Registry ACTRN12623000473662 registered 09/05/2023.Approved by UniSC Human Research Ethics Committee (A221828) and the UK parkrun Research Board. Original protocol. Authors SB, RB, HHW, MM, YK.

**Supplementary Information:**

The online version contains supplementary material available at 10.1186/s13102-024-00882-w.

## Background

Regular exercise for cancer survivors is strongly recommended to reduce the severity of adverse treatment effects, new cancer onset and cancer-specific and all-cause mortality [1]. Cancer survivors are patients living with cancer, either undergoing medical treatment or in remission. Between 60 and 70% of survivors do not meet the recommended levels of aerobic physical activity (PA) and nearly 90% do not meet strength training recommendations [1, 2]. The Clinical Oncology Society of Australia Position Statement on Exercise in Cancer Care [1] includes recommendations that individuals should aim towards, and maintain, weekly participation in at least 150 min of regular moderate-intensity aerobic exercise, or 75 min of more vigorous higher-intensity aerobic exercise. Strength training is also recommended two to three times per week [3, 4]. The reasons why cancer survivors are not meeting the physical activity levels are still somewhat unclear. Previous research with breast and prostate cancer patients suggests that reasons for low exercise participation rates include access issues, poor levels of baseline fitness, medical appointments and treatments, disease symptoms and treatment side effects [5–10]. These issues may also be relevant for other types of cancers. However, useful strategies for improving PA uptake and adherence have not been robustly investigated.

To prescribe appropriate, sustainable exercise for cancer survivors, we need to understand the factors that influence enjoyment and acceptance of, and adherence, to PA. Growing evidence from group exercise programs shows that social connection and support play a big role in exercise adherence [11, 12]. The established global run and walk event, parkrun, is a free community-based walk or run of 5 km, conducted in selected local parks [13, 14]. The event is monitored by volunteers who check that all participants have completed the course or have withdrawn safely. Parkrun has been endorsed by general practitioners as a way of preventing and managing chronic lifestyle conditions such as cardiac disease, hypertension and mental health conditions [13–15]. Recent research has shown that parkrun is effective for improving fitness and health in sedentary non-clinical populations, and also for individuals with heart disease and obesity [16–18]. The structure of parkrun suggests that it is a mode of physical activity that appeals to people with low fitness levels, and it provides a framework and social support that can be beneficial for people with chronic diseases and disabilities [19–21]. There is evidencefrom recent studies that parkrun participants reported more benefits from, and motivation for, parkrun as an activity, compared to other forms of exercise because it enhanced their happiness and satisfaction, they found it a real achievement, they met new people, spent more time outdoors, gained more social connections, felt part of a community and engaged more with friends who also did parkrun [18–20]. Parkrun can be self-paced and therefore has the potential to be a manageable, safe mode of exercise for individuals with low functional capacity [21]. Despite addressing many of the barriers to PA, parkrun has yet to be trialed as a mode of exercise rehabilitation for cancer survivors.

This project will investigate the feasibility of parkrun as a mode of physical activity for cancer survivors, with regard to acceptability, enjoyment and social identification for participants, and efficacy in maintaining or improving physical and functional status, and health-related quality of life (HRQoL) [16, 19, 20]. This study may provide much-needed evidence that parkrun is accessible, inclusive, supportive, safe and effective, and that it can provide real benefits for patients that can be translated to clinical practice [21, 22]. 

## Methods

### Experimental design

This feasibility study is ethically-approved (A2218280 and registered with Australian and New Zealand Clinical Trials Registry 9th May 2023 (identifier: ACTRN12623000473662) with recruitment about to start. No data has been collected and no results published. The manuscript is in accordance with SPIRIT guidelines (http://www.spirit-statement.org/spirit-statement/) for the reporting of study protocols (Table 1) and adheres to the CONSORT guidelines.

**Table 1:** Outline measures and timeline of the study

	Enrolment	Allocation	Post Allocation				
TIMEPOINT			Baseline T1	Control Block T2	Intervention Block T3	Follow-up T4	Trial End Point
			0	4 weeks	End of 6 month parkrun block	2 months post-intervention	Post - T4
ENROLMENT:	X						
Eligibility screen	X						
Informed consent	X						
Allocation	X						
Single Group Own Controls		X					
**Assessments / Tools**							
Demographics & Clinical Characteristics			X				
Physical Function (Resting HR, BP, O2sat, 6MWT, 30-s Sit-to-Stand)			X	X	X		
Health-related Quality of Life (EORTC QLQ-30) questionnaire			X	X	X	X	
Anxiety & Depression (HADS) questionnaire			X	X	X	X	
General Physical Activity (IPAQ-SF)			X	X	X	X	
Dietary Intake (MEDAS) questionnaire			X	X	X	X	
Mood (Daylio app)			X	X	X	X	
Sleep Quality (Daylio app)			X	X	X	X	
Participant Satisfaction, Diet, Exercise behaviour, Intent Feedback Surveys					X	X	
Parkrun event Adherence					X	X	
INTERVENTIONS:							
Parkrun participation					X		

**Fig. 1:**
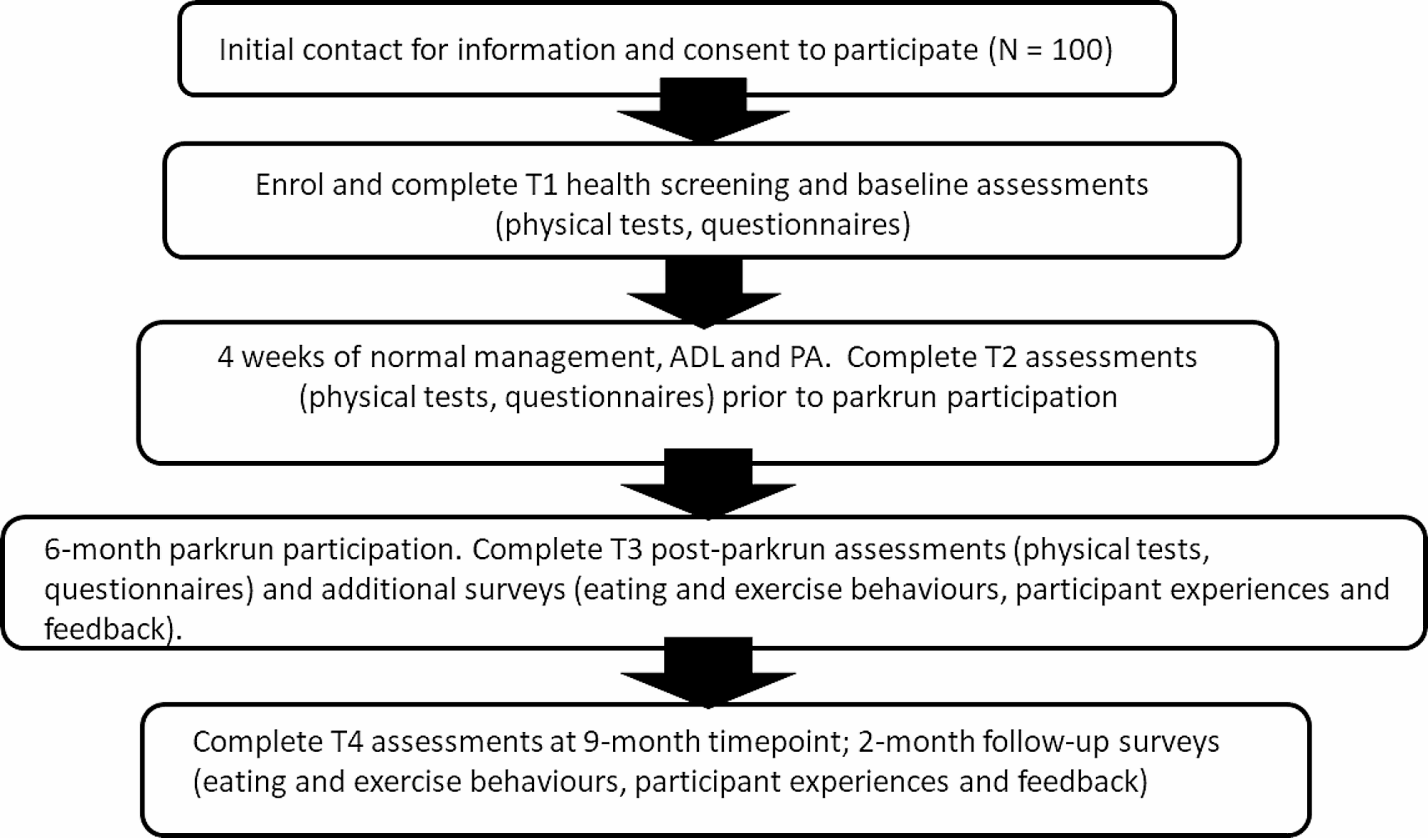
Study flowchart,Abbreviations: ADL, activities for daily living; PA, physical activity

This protocol describes a single group cohort study with participants acting as their own controls [23]. The study outline and timeline is shown in Fig. 1.

We will recruit 100 cancer survivors over the age of 18, during a period of 24 months. The study will be advertised using the University of the Sunshine Coast (UniSC) news webpage, local and state cancer support groups, local parkrun sites, local oncologists, cancer care nurses and allied health professionals, radio and other media, newsletters and fliers, and relevant social media platforms. The initial recruitment phase will be for 6 weeks but we anticipate an ongoing recruitment strategy through 2024. Potential participants can contact the researchers for further information and can be sent the information sheet and a consent form. Informed consent must be given prior to study enrolment.

### Participants

All participants will be provided with a project information sheet detailing the project aims and structure and outlining the time commitment. Written consent must be given to the researchers prior to study enrolment and participation. Participants must also register with parkrun and receive their ID number prior to intervention participation. The informed consent process will continue through the study as the researchers and participants affirm procedures at each contact point of data collection. Participants are free to withdraw at any time.

### Study sites

Participant screenings, pre- and post-intervention exercise assessments and some questionnaires will be completed at the University of the Sunshine Coast Exercise Science laboratories. The parkrun events for the 6-month intervention are offered at nine parks on the Sunshine Coast: Mudjimba, Brightwater, Baringa, Harmony, Kawana, Golden Beach, Nambour, Maleny, Noosa. (Map, Supplementary file 3; park route information via parkrun Australia website: https://www.parkrun.com.au/)

### Inclusion criteria

Cancer survivors, aged 18 years or over, diagnosed with any type of cancer (e.g. breast, prostate, colorectal, blood, bone), who have medical clearance to walk and exercise, are eligible for the study. “Cancer survivor” is described as undergoing medical treatment or in remission. Participants would be classified according to the Palliative Care Outcomes Classification (PCOC); either Stage 1 (stable) or Stage 2 (unstable but not deteriorating) [24]. Participants must have given informed consent and must be registered with parkrun before start of the first walk/run event. Participants must be able to understand and communicate in English or participate with a carer/support person who can communicate in English. Finally, participants must be able to commit to the 9-month period of the study.

### Exclusion criteria

Cancer survivors are ineligible for this study if they are unable to walk, are classified by the PCOC as Stage 3 (deteriorating) or 4 (terminal) or are undergoing end-of-life care, and if they do not have medical clearance to at least walk or do other light exercise. Potential participants will also be excluded if they have a serious medical condition such as uncontrolled cardiac disease (e.g. angina, arrythmias, heart failure) [25] and/or hypertension; serious pulmonary disease where forced vital capacity (FVC) is less than 1 L; uncontrolled metabolic or renal disease; a current musculoskeletal injury that might cause pain during walking or jogging; a neurological condition that is a falls or exercise risk for participants; severe visual, and/or auditory impairment, or behavioural, cognitive or psychological disorder, that would affect understanding and complying with clear instructions or communicating with others. Patients are ineligible for the study if they cannot communicate in, or understand, English or follow verbal instructions from parkrun volunteers and/or a carer/support person who can speak English.

### Intervention

The study has a participant time commitment of 9 months, which includes 1 month of normal activities and management to establish baseline values for outcome measures, followed by 6 months of parkrun participation, and then a follow up at 2 months post-intervention. During the 6 months of parkrun, participants can complete events at their discretion. Parkrun events occur each Saturday morning but we anticipate that many participants may not be able to join in on a weekly basis due to cancer treatment cycles, symptoms, family or work commitments or poor weather. Parkrun events start at approximately 7am, and participants can walk or jog at their own pace. There is no set finish time but the events are normally completed within 2 h, allowing social gathering time afterwards.Volunteer marshals are stationed every few hundred metres along the paths to assist with directions, encouragement and for participant safety. Each participant wears their individual barcode so that their finish time is recorded electronically, captured and stored by the parkrun organisation. Individual results can be accessed by each participant.Volunteers have first aid and CPR training.Each course will have a first aid kit and automated defibrillator available and access for emergency vehicles if necessary.In the case of bad weather, an event would be cancelled.

## Outcomes

### Study objectives

This feasibility study has been developed primarily to assess the acceptability of, and adherence to, parkrun as an enjoyable exercise intervention for cancer survivors. Secondary aims are to assess the efficacy of parkrun to improve social support, HRQoL, mental health, to prevent physical and functional decline [3, 4, 26] as well as influence dietary intake [26–28]. 

We hypothesised that:

1. Consistent parkrun participation will improve cancer survivor engagement in physical activity, social interaction and social identity.
2. Cancer survivors will enjoy doing parkrun events.
3. Parkrun participants will have improved physical function, HRQoL, mental health, disease symptoms and treatment side-effects compared to baseline measures.
4. Parkrun participation may indirectly affect the dietary intake of cancer survivors.

Outcome measures will be assessed at enrolment and initial screening [25] (baseline, T1); after 4 weeks of usual management and activities of daily living (ADL, T2); after 6 months of parkrun participation (T3); 2 months post-intervention follow-up (T4) (Fig. 1).

### Demographics and clinical characteristics

At T1 the following data will be recorded: participant age, gender, cancer type and stage, time since diagnosis, PCOC status, type of referral to the study (e.g. medical practitioner, media, word of mouth), symptoms, types of treatment and side effects, relevant allied health management and other existing medical conditions if applicable. We will also record height, weight, body mass index (BMI), resting heart rate and blood pressure.

### Outcome measures

1. *Physical function*: Resting heart rate, oxygen saturation (recorded using a pulse oximeter), resting blood pressure (standard auscultation), 6 min Walk Test (6MWT) and 30 s Sit-to-Stand test. These measures will be recorded at T1, T2 and T3. Participant rating of perceived effort (RPE) [29] will be recorded during and after the 6MWT and Sit-to-Stand test.
2. *Quality of Life*: Assessed using the European Organization for Research and Treatment of Cancer (EORTC) Quality of Life Questionnaire (QLQ-30) [30]. This is a validated 30-question instrument used to assess different aspects contributing to HRQoL in cancer patients. Responses will be recorded at T1-T4.
3. *Anxiety and depression*: Measured with the Hospital Anxiety and Depression Scale (HADS) [31]. This validated questionnaire consists of seven questions scoring levels of anxiety and seven assessing levels of depression. The scale takes approximately three to five minutes to complete and will be used at T1-T4.
4. *GENERAL physical activity*: General physical and incidental activity, including ADL, and sitting time, will be assessed using the validated Short Form International Physical Activity Questionnaire (IPAQ-SF) [32] at T1-T4.
5. *Dietary intake*: The Mediterranean Diet Adherence Screener (MEDAS) [33, 34] is a validated dietary assessment tool to determine adherence to a Mediterranean diet, which has been associated with improved health outcomes of cancer survivors. It consists of 14 questions (yes/no response) to assess the frequency of food groups included in the Mediterranean diet and will be administered at T1-T4.
6. *Mood and sleep*: The Daylio Mood Diary basic version app is freely available for download to phone or smartwatch. The app allows daily diary entries for mood and sleep quality, and produces weekly and monthly graphs to track diary inputs. The app also allows participants to tap on icons that may contribute to mood and sleep quality e.g. food and water intake, stress, hobbies and leisure pursuits. These may contribute to parkrun adherence. Monthly graphs will be exported to the research team’s contact email by each participant, and data collated for each time point (T1-T4).
7. *Participation satisfaction, diet and exercise behaviour, and intent*: A participant survey will be administered at T3 and T4. This consists of a checklist about eating and exercise behaviours, and participant feedback about the parkrun project during the study and 2-month follow up, including intent to change behaviours. The survey also includes a series of open-ended questions about parkrun where participants are encouraged to provide their opinions, likes and dislikes, levels of enjoyment, barriers to involvement, whether the intervention provided benefits and social support, and intention to continue with parkrun or other physical activity. For example, specific questions ask whether participants find parkrun fun and acceptable as rehabilitation exercise; whether it is easy to join in; did they meet new people (including other cancer survivors) and make new social connections; if they feel supported and incentivised to keep coming; if they feel safe participating; and is their quality of life improved.
8. *Attendance and adherence*: Parkrun can provide the number of events attended by each participant through their national database, using the individual’s ID barcode number. Adherence will be calculated by dividing the number of events attended by each person by the number of available events in the 6-month period of the intervention and multiplying by 100 to give a percentage. If a participant does not finish an event, their time is not recorded.

### Patient and public involvement

The study protocol underwent an external review for objective feedback prior to HREC submission. The project aims and measures were also discussed with a panel of external providers, health professionals, including an oncologist, and with a small group of cancer survivors who would not be taking part in the study.

### Statistical analyses, sample size and power calculation

Numerical data from demographic, physical and scored questionnaire outcome measures will be analysed using Excel spreadsheets, IBM©SPSS version 29 and R studio [35]. Qualitative data (mood, participant feedback survey responses, participants’ attitudes towards the intervention, enjoyment, appropriateness, suitability, convenience and perceived effectiveness of the intervention) will be analysed thematically using NVivo and following the process of Braun and Clarke (2006) [36]. Comments will be read, organised and coded using an iterative framework (familiarisation; generation of initial codes; search for themes; review of themes; definition of themes and a final report). The codes will be reviewed, discussed and refined with the coding framework drawn from the data as well as being informed by the survey questions and themes highlighted in wider literature on parkrun outcomes [19, 20]. 

Participant demographic and cancer-related variables will be summarised using descriptive statistics (e.g. count or percent; mean and standard deviation). Linear mixed effect models will be used to determine the effect of parkrun participation on the proportion of participants meeting minimal PA recommendations. These models will include a random intercept for each participant, to account for the clustering of observation on each individual (i.e. measurements at baseline, 4-weeks usual activities, following 6 months of parkrun participation, and 2-month follow-up). A histogram and quantile-quantile plot of the model residuals will be used to determine whether the assumption of normally distributed errors is met. Model fit will be assessed using the adjusted R^2^ value and the model residual standard error; the most parsimonious model will be selected for inferences. Effect sizes (Cohen’s d) will be calculated where there are significant effects of time using the pooled standard deviation as the denominator.

In the case of participant attrition from the study, or if participants miss parkrun events in the 6-month intervention period, researchers will follow up with each participant individually, either through the study or at the end of the study. Reasons for reduced attendance or attrition can be gained from the participant and this information is valuable for informing the researchers of potential barriers or issues with parkrun as a mode of cancer rehabilitation exercise.

Sample size was determined via simulation methods using a random intercept model with the R package [35]. We were interested in powering the study to detect changes in exercise adherence (measured as the proportion of cancer patients meeting minimum physical activity guidelines) and used this outcome variable in simulations. Data used to create the model were informed by previous studies [8, 10]. We assumed the average proportion of cancer patients meeting minimal physical activity guidelines for aerobic exercise was 30% [37]. Time (three levels- baseline, 6-months parkrun and follow-up), body mass index (BMI) and sex (two levels- male and female) were included as fixed factors within the sample size model. A random intercept was included for each individual participant. BMI and sex were included as fixed factors as these variables can influence exercise adherence in cancer patients [8–10]. Calculations were made assuming fixed effects of 6%, 30% and 5% for sex, time and BMI respectively, and a standard deviation of the random intercept of 2%. Based on these assumptions and a type I error rate of 5%, 80 participants give approximately 80% power to detect the specific time effect of 30% increase in the proportion of participants meeting physical activity recommendations. A minimum of 100 participants will be recruited, which even allowing for potential attrition of 20 participants would still provide a minimum sample size of 80.

### Data management

All data will be given a numerical ID (parkrun ID number) and will be de-identified prior to any analysis. Participant contact details will be kept confidential and used only for points of contact regarding project enolment, data collection appointments and emailing/posting of paperwork. Participant details will only be available to the research team.

SB, RB, SC, HHW and YK will be in charge of data management. Electronic data will be stored in a project-specific, password-protected external hard drive and all backed up, de-identified data will be stored on the protected USC R drive. Any written material will be locked in a filing cabinet in the lead researcher’s office. We plan to make de-identified data and analysis code available in public repositories. All data will remain confidential and will be not identifiable for purposes of statistical analyses and publication. Coded data from each participant will be used for quantitative and qualitative anlyses. De-identified data and analysis codes can be made available to external researchers upon email request to the lead researcher. All data will be stored for a minimum of 5 years from the completion of the research as per university research guidelines and procedures. After archiving for a minimum of 5 years, any hard copy material may be shredded using contracted service providers. Project progress will be monitored throughout the duration of the study, with annual reporting to the USC Human Ethics Committee, and with monthly reporting to the research team members and project advisors.

### Dissemination

Deidentified study results will be published in peer-reviewed journals and at appropriate conferences. Summarized study results will also be sent to participants and to interested cancer-support organisations. A summary report will also be sent to the United Kingdom parkrun organization, who approve for any international parkrun research and request a summary of final results for publication on their website. If the research team receive external funding, a summary report will need to be sent the funding organization as part of the funding agreement.

## Discussion

Regular physical activity for people living with and beyond cancer, including aerobic, strength and other modes of exercise, are strongly recommended by oncologists, medical practitioners and exercise professionals. Exercise is regarded as an adjuvant treatment for cancers and can reduce some symptoms and treatment side effects. However, the majority of cancer survivors are not meeting recommended levels of exercise. The reasons are still unclear, are likely to be varied, and require further research. To improve strategies for exercise adherence, we need to understand more about patient enjoyment and acceptance of exercise modalities, and potential barriers to exercise participation. Previous studies have noted that group and community-based exercise programs can be successful because they provide a social framework and support, and companionship. This study will investigate the feasibility of using parkrun, an existing free group walk-run event, to engage cancer survivors in regular, self-paced walking or jogging.

### Limitations

The study has several strengths and limitations. Advantages include the use of an existing exercise program that has no direct costs, is easily accessible, is outdoors, and is supportive and safe in nature. The study will comprehensively profile participants and will have quantitative and qualitative outcome measures, participants’ experiential data and follow-up surveys. Limitations include the lack of randomisation and inclusion of participants with different types and stages of cancers. However, using a heterogenous sample is useful from the perspective of real-world clinical practice. As a feasibility study, this trial aims to identify benefits and barriers that affect parkrun participation and participant enjoyment and acceptance, and to lead to a larger longitudinal study.

### Electronic supplementary material

Below is the link to the electronic supplementary material.

Supplementary Material 1

Supplementary Material 2

Supplementary Material 3

Supplementary Material 4

## Data Availability

Datasets to be analysed in the study will be available from the corresponding author on reasonable request.

## Declarations

### Patient and public involvement

Patients, an oncologist and external providers were consulted in the development of this project.

### Provenance and peer review

Not commissioned; externally peer-reviewed.

## Abbreviations

ADL: activities of daily living
PA: physical activity
HRQoL: health-related quality of life
PCOC: Palliative Care Outcomes Classification
FVC: forced vital capacity
BMI: body mass index
6MWT: Six Minute Walk Test
RPE: rate of perceived exertion
EORTC QLQ-30: European Organization for Research and Treatment of Cancer Quality of Life Questionnaire
HADS: Hospital Anxiety and Depression Scale
IPAQ-SF: International Physical Activity Questionnaire Short Form
MEDAS: Mediterranean Diet Adherence Screener
