# Supplementary material for: Study protocol - assessing parkrun for walking rehabilitation for people living with, and beyond, cancer: acceptability, adherence, social support and physical function
Source: BMC Sports Sci Med Rehabil. 2024 Apr 19;16:88. doi: 10.1186/s13102-024-00882-w (PMC11027354; doi:10.1186/s13102-024-00882-w)
Supplement: Supplementary file 2 — Supplementary Material 2 [file 13102_2024_882_MOESM2_ESM.docx]

**
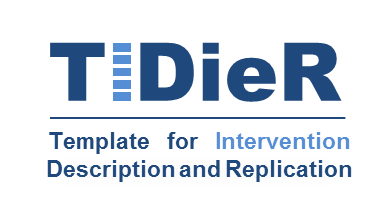
The TIDieR (Template for Intervention Description and Replication) Checklist*:**

Information to include when describing an intervention and the location of the information

| **Item number** | **Item** | **Where located **** | |
| --- | --- | --- | --- |
|  |  | Primary paper  (page or appendix  number) | Other ^†^ (details) |
|  | **BRIEF NAME** |  |  |
| **1.** | *Provide the name or a phrase that describes the intervention.*  Assessing parkrun for walking rehabilitation for cancer survivors: a 6-month intervention | Page 1, 4 | . |
|  | **WHY** |  |  |
| **2.** | *Describe any rationale, theory, or goal of the elements essential to the intervention*.  This project will investigate the feasibility of parkrun as a mode of physical activity for cancer survivors, with regard to acceptability, enjoyment and social identification for participants, and efficacy in maintaining or improving physical and functional status, and health-related quality of life. Most cancer survivors do not meet current guidelines for adequate physical activity and this group exercise mode may encourage survivors to be more active, given the published benefits of parkrun for other chronic conditions. | Page 4, 5 |  |
|  | **WHAT** |  |  |
| **3.** | *Materials: Describe any physical or informational materials used in the intervention, including those provided to participants or used in intervention delivery or in training of intervention providers. Provide information on where the materials can be accessed (e.g. online appendix, URL).*  Participants will be shown how to access parkrun online to register for their ID barcode by the research team, and will be advised on the location of specific local parks used for each event. Verbal advice on participation risks and benefits, and appropriate clothing, footwear, and sun protection will be given before the start of the intervention block. | Page 7  Page 13 Study Protocol at ANZCTR URL https://www.anzctr.org.au/ |  |
| **4.** | *Procedures: Describe each of the procedures, activities, and/or processes used in the intervention, including any enabling or support activities*.  **Procedures:** Participants will complete an online parkrun registration within the 4 week “Control” period, prior to the start of the 6-month parkrun intervention. Once registered, parkrun emails each participant an individual barcode ID number, which they present to parkrun officials at the start of every parkrun event. The ID allows each person to access their event finish time online, and allows the researchers to count the number of events each participant completes in the 6 months. Participants can complete some or all of the parkrun course at their own pace; each event has volunteers stationed along the course every few hundred metres for safety and to encourage participants. Some of the research team also do parkrun events and can meet up with participants at times, if they attend the same events; this will help with adherence and compliance, and social connectivity.  **6-month intervention: Parkrun group events involve self-paced walking or jogging as tailored by each participant around set 5km courses at specific parks.** | Page 8 - 10 |  |
|  | **WHO PROVIDED** |  |  |
| **5.** | *For each category of intervention provider (e.g. psychologist, nursing assistant), describe their expertise, background and any specific training given.*  Accredited clinical exercise physiologists, an exercise scientist and a dietician will conduct the outcome measure assessments. Parkrun events are run by the organisation’s marshalls and volunteers. | N/A |  |
|  | **HOW** |  |  |
| **6.** | *Describe the modes of delivery (e.g. face-to-face or by some other mechanism, such as internet or telephone) of the intervention and whether it was provided individually or in a group.*  Individual participation in a group intervention | Page 4, 5, 7 |  |
|  | **WHERE** |  |  |
| **7.** | *Describe the type(s) of location(s) where the intervention occurred, including any necessary infrastructure or relevant features*.  Sunshine Coast parkrun locations are parks at Mudjimba, Brightwater, Baringa, Kawana, Harmony, Golden Beach, Nambour, Maleny and Noosa. The event courses are manned by marshals and volunteers stationed regularly for directions, safety and encouragement. Each course will have a first aid kit and automated defibrillator available and access for emergency vehicles if necessary. | Page 5 |  |
|  | **WHEN and HOW MUCH** |  |  |
| **8.** | *Describe the number of times the intervention was delivered and over what period of time including the number of sessions, their schedule, and their duration, intensity or dose.*  The parkrun intervention will run for 6 months for each participant. The parkrun events run weekly throughout the year beginning at 7am Saturdays in specific local parks. Participants can select which event they want to attend. The session time depends on how fast each participant walks or jogs, and whether they wish to complete the entire 5km distance. Participants will be encouraged to attend as many events as possible but if unwell or affected by poor weather, family or other issues, they are not compelled to attend. Walk or run speed is self-determined. | Page 5, 7 |  |
|  | **TAILORING** |  |  |
| **9.** | *If the intervention was planned to be personalised, titrated or adapted, then describe what, why, when, and how.*  Participants can tailor the intervention if needed by (1) attending parkrun events when their symptoms allow; (2) choosing their walk speed to suit their symptoms and fitness levels; (3) using the option of not completing the full 5km event distance if they wish | Page 7, 9, 14 |  |
|  | **MODIFICATIONS** |  |  |
| **10.^ǂ^** | *If the intervention was modified during the course of the study, describe the changes (what, why, when, and how).*  The intervention will not be modified | N/A |  |
|  | **HOW WELL** |  |  |
| **11.** | *Planned: If intervention adherence or fidelity was assessed, describe how and by whom, and if any strategies were used to maintain or improve fidelity, describe them.*  When underway, intervention adherence will be monitored through use of event attendance data which is stored in deidentified format by the parkrun organisation. We will calculate adherence as a study outcome. | Pages 8, 11, 13 |  |
| **12.^ǂ^** | *Actual: If intervention adherence or fidelity was assessed, describe the extent to which the intervention was delivered as planned*.  Intervention has not begun | N/A |  |

** **Authors** - use N/A if an item is not applicable for the intervention being described. **Reviewers** – use ‘?’ if information about the element is not reported/not sufficiently reported.

† If the information is not provided in the primary paper, give details of where this information is available. This may include locations such as a published protocol or other published papers (provide citation details) or a website (provide the URL).

ǂ If completing the TIDieR checklist for a protocol, these items are not relevant to the protocol and cannot be described until the study is complete.

* We strongly recommend using this checklist in conjunction with the TIDieR guide (see *BMJ* 2014;348:g1687) which contains an explanation and elaboration for each item.

* The focus of TIDieR is on reporting details of the intervention elements (and where relevant, comparison elements) of a study. Other elements and methodological features of studies are covered by other reporting statements and checklists and have not been duplicated as part of the TIDieR checklist. When a **randomised trial** is being reported, the TIDieR checklist should be used in conjunction with the CONSORT statement (see [www.consort-statement.org](http://www.consort-statement.org)) as an extension of **Item 5 of the CONSORT 2010 Statement.** When a **clinical trial** **protocol** is being reported, the TIDieR checklist should be used in conjunction with the SPIRIT statement as an extension of **Item 11 of the SPIRIT 2013 Statement** (see [www.spirit-statement.org](http://www.spirit-statement.org)). For alternate study designs, TIDieR can be used in conjunction with the appropriate checklist for that study design (see [www.equator-network.org](http://www.equator-network.org)).
