# Supplementary material for: Study protocol - assessing parkrun for walking rehabilitation for people living with, and beyond, cancer: acceptability, adherence, social support and physical function
Source: BMC Sports Sci Med Rehabil. 2024 Apr 19;16:88. doi: 10.1186/s13102-024-00882-w (PMC11027354; doi:10.1186/s13102-024-00882-w)
Supplement: Supplementary file 4 — Supplementary Material 4 [file 13102_2024_882_MOESM4_ESM.docx]

**SUPPORTING INFORMATION**

Supplement 1 SPIRIT Checklist

Supplement 2 TIDier Checklist

Supplement 3 Map of parkrun locations
